# Crucial Role of Lysine-Specific Histone Demethylase 1 in RANKL-Mediated Osteoclast Differentiation

**DOI:** 10.3390/ijms24043605

**Published:** 2023-02-10

**Authors:** Mina Ding, Zhihao Chen, Eunjin Cho, Sang-Wook Park, Tae-Hoon Lee

**Affiliations:** 1 BioMedical Sciences Graduate Program (BMSGP), Chonnam National University, Gwangju 61186, Republic of Korea; 2 Department of Oral Biochemistry, Dental Science Research Institute, School of Dentistry, Chonnam National University, Gwangju 61186, Republic of Korea

**Keywords:** osteoporosis, osteoclast differentiation, LSD1, small molecule

## Abstract

Epigenetic regulators are involved in osteoclast differentiation. This study proposes that the inhibitors of epigenetic regulators could be effective in the treatment of osteoporosis. This study identified GSK2879552, a lysine-specific histone demethylase 1 (LSD1) inhibitor, as a candidate for the treatment of osteoporosis from epigenetic modulator inhibitors. We investigate the function of LSD1 during RANKL-induced osteoclast formation. LSD1 small-molecule inhibitors effectively inhibit the RANKL-induced osteoclast differentiation in a dose-dependent manner. LSD1 gene knockout in macrophage cell line Raw 264.7 also inhibits RANKL-mediated osteoclastogenesis. LSD1-inhibitor-treated primary macrophage cells and LSD1 gene knockout Raw 264.7 cells failed to show actin ring formation. LSD1 inhibitors prevent the expression of RANKL-induced osteoclast-specific genes. They also downregulated the protein expression of osteoclast-related markers in osteoclastogeneses, such as Cathepsin K, c-Src, and NFATc1. Although LSD1 inhibitors were shown to reduce the in vitro demethylation activity of LSD1, they did not modulate the methylation of Histone 3 K4 and K9 during osteoclastogenesis. The ovariectomy (OVX)-induced osteoporosis model revealed that GSK2879552 slightly restores OVX-induced cortical bone loss. LSD1 can be employed as a positive regulator to promote osteoclast formation. Hence, inhibition of LSD1 activities is a potential target for preventing bone diseases characterized by excessive osteoclast activities.

## 1. Introduction

The balance between bone formation by osteoblasts and bone resorption by osteoclasts regulates bone mass [1,2]. Excessive osteoclast activity causes bone imbalance, which leads to many diseases, including osteoporosis, bone tumors, Paget’s disease, and rheumatoid arthritis (RA) [3]. Therefore, it is imperative to identify new therapeutic targets for the treatment of osteoporosis and develop novel anti-osteoclastic activity agents.

Differentiation of osteoclasts is induced by two essential factors: macrophage-colony stimulating factor (M-CSF) and receptor activator of nuclear factor kappa B (NF-κB) ligand (RANKL) [4]. When RANKL binds to RANK, tumor necrosis factor receptor-associated factor 6 (TRAF6)—a splice molecule—is recruited, which leads to the activation of NF-κB and mitogen-activated protein kinase (MAPK) signaling pathways, ultimately activating the nuclear factor of activated T cells 1 (NFATc1) [5]. NFATc1 plays a major role in the regulation of several osteoclast-specific genes, including matrix metallopeptidase 9 (*MMP9*), Cathepsin K (*Ctsk*), and acid phosphatase 5, tartrate resistant (*ACP5*) [6,7]. Additionally, c-fos, a transcription factor, has been identified as a positive regulator of RANKL-induced osteoclast formation [8]. It has been reported that c-Src kinase is required for osteoclast bone-resorbing activity [9].

Epigenetics is generally described as a genomic mechanism that reversibly regulates gene activity without altering the DNA sequence [10]. Epigenetic changes alter the DNA structure by modifying DNA replication and/or causing post-translational modifications of DNA-associated proteins [11]. Some recent studies have shown that epigenetic regulators perhaps already play a critical role in osteoclast differentiation [12]. Because epigenetic regulators are involved in osteoclast-mediated bone resorption and bone formation, we hypothesize that the inhibitors of epigenetic regulators could be effective in the treatment of osteoporosis. Therefore, we herein focus on identifying a novel pharmacological agent from epigenetic inhibitors to increase the treatment possibilities for osteoporosis.

In histone methylation, one, two, or three methyl groups are transferred to certain amino acids of histones, such as lysine, arginine, and histidine. Histone methylation is a dynamic process, in which methyl moieties can be added or removed by specific enzymes [13,14], which are involved in tumorigenesis [15], and angiogenesis [16], as well as in the development of acute myeloid leukemia (AML) [17]. Histone methylation is regulated by histone methyltransferases and histone demethylases, and these methyl-modifying enzymes are involved in the regulation of bone cell differentiation [18]. Demethylation of lysine-specific histone demethylase 1 (LSD1) of histone H3 lysine 4 is linked to gene repression, whereas demethylation of histone H3 lysine 9 results in gene activation [19]. LSD1 has also been shown to demethylate non-histone proteins and regulate their cellular functions [20]. LSD1 interacts with p*53* to attenuate p53-mediated transcriptional activation by demethylating K370 of HEK-293 cells [21]. LSD1 has also been shown to be phosphorylated by PKCα, which promotes p65 demethylation and enhances p65 protein stability in the inflammatory response [22]. LSD1 directly binds to CoREST (RCOR1) by the extended tower domain which affects its function [23]. Numerous studies have demonstrated that the presence of LSD1 and its function play important roles in mammalian development, the establishment and maintenance of stemness, and the progression of cancer [24]. Furthermore, several studies have strongly indicated that LSD1 is involved in the differentiation of several cell types such as adipogenesis [25] and muscle stem cells [26], as well as in the epithelium-to-mesenchyme transition [27]. Inhibition of LSD1 promotes osteoblast differentiation and bone formation in human mesenchymal stem cells (hMSCs) [28]. Interestingly, Petri et al. reported that the suppression of the LSD1 activity inhibits osteoblast differentiation [29]. A recent study indicated that the deletion of LSD1 in female mice increases bone mass [30]. The involvement of LSD1 in the regulation of bone mass has also been reported; however, the role of LSD1 in RANKL-induced osteoclast differentiation in murine macrophages has not yet been elucidated.

Because LSD1 has a complex role in the regulation of bone remodeling, we hypothesize that it could regulate RANKL-induced osteoclast differentiation of the mouse bone marrow. Thus, the present study explores the role of LSD1 in RANKL-induced osteoclastogenesis through the pharmacological inhibitors of LSD1 by small-molecule inhibitors or knockout of the LSD1 gene in Raw 264.7 cells. We selected GSK2879552 as the candidate for the treatment of osteoporosis from epigenetic modulator inhibitors. GSK2879552 and GSK-LSD1 are structurally similar and selective irreversible inactivators of LSD1 [31]. Thus, we also employ another LSD1 inhibitor, GSK-LSD1, to investigate the effects of the LSD1 inhibitor on osteoclast formation. Additionally, this study investigates the effect of knockout of LSD1 on the differentiation of osteoclasts in Raw 264.7 cells. Finally, we investigated whether LSD1 is a potential target for the treatment of diseases associated with excess osteoclast activity.

## 2. Results

### 2.1. Identification of Anti-Osteoporosis Candidates from Epigenetic Modulator Inhibitors

**Figure 1:**
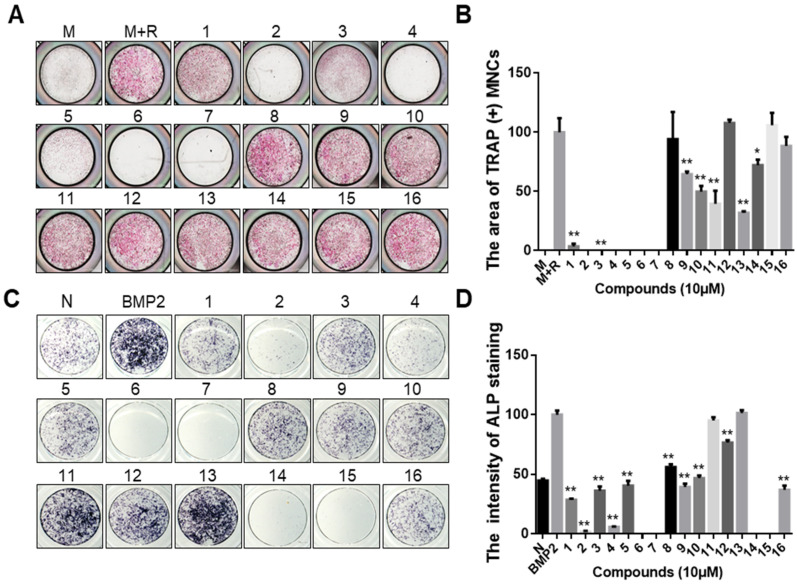
Effect of epigenetic regulator inhibitors on osteoclast differentiation and osteoblast differentiation. (**A**) BMMs were treated with M-CSF (30 ng/mL) and RANKL (50 ng/mL), and then incubated with or without epigenetic regulators (10 μM). After 4 days, the cell was fixed and stained for the TRAP assay. (**B**) Quantitative analysis of the area of TRAP-positive multinucleated osteoclasts [TRAP (+) MNCs] by Image J software. M: M-CSF, M+R: M-CSF + RANKL. * *p* < 0.05, ** *p* < 0.01 versus the control (M+R) group. (**C**) Primary calvarial pre-osteoblast cells were treated with BMP2 (100 ng/mL) and co-cultured with or without epigenetic regulators (10 μM) for 7 days. The differentiation cell was stained with ALP. (**D**) Quantified analysis of ALP staining intensity by Image J software. “N” indicates no BMP2-treated group. ** *p* < 0.01 versus the BMP2 treatment group. The structures of the compounds corresponding to these numbers are shown in Table S1.

A primary screen was constructed among 16 epigenetic modulation inhibitors to select candidates as putative osteoporosis-regulating small molecules (Table S1). To identify whether epigenetic regulator inhibitors can effectively inhibit RANKL-induced osteoclast differentiation, epigenetic regulator inhibitors were treated with murine bone marrow-derived macrophages (BMMs). We first measured the formation of mature osteoclasts after treating the cells with or without epigenetic regulator inhibitors by tartrate-resistant acid phosphatase (TRAP) staining. The results suggested that compounds 1, 3, 9, 10, 11, and 13 in Table S1 significantly inhibited osteoclast differentiation, compound 14 slightly inhibited osteoclast differentiation, whereas compounds 2, 4, 5, 6, and 7 had significant cytotoxic effects on osteoclast precursor cells at 10 µM (Figure 1A,B). The name and structures of the compounds are shown in Table S1. In addition, we investigated the effect of epigenetic modulator inhibitors on osteoblastogenesis by conducting an alkaline phosphatase (ALP) staining assay using murine calvarial pre-osteoblast cells. The results of ALP staining and quantification analysis are shown in Figure 1C,D. From an osteoblastogenesis point of view, compounds 11 and 13 did not affect osteoblast differentiation below 10 μM concentration, whereas compounds 1, 3, 5, 8, 9, 10, 12, and 16 suppressed BMP2-induced osteoblast differentiation. In addition, compounds 2, 4, 6, 7, 14, and 15 had cytotoxic effects on pre-osteoblast cells at 10 µM. Compound 13 inhibited OC differentiation more effectively than compound 11 at low concentrations. Therefore, compound 13 (GSK2879552) was selected as a candidate for the treatment of osteoporosis from epigenetic modulator inhibitors.

### 2.2. LSD1 Inhibitors Prevent RANKL-Stimulated Osteoclast Differentiation in a Dose-Dependent Manner

**Figure 2:**
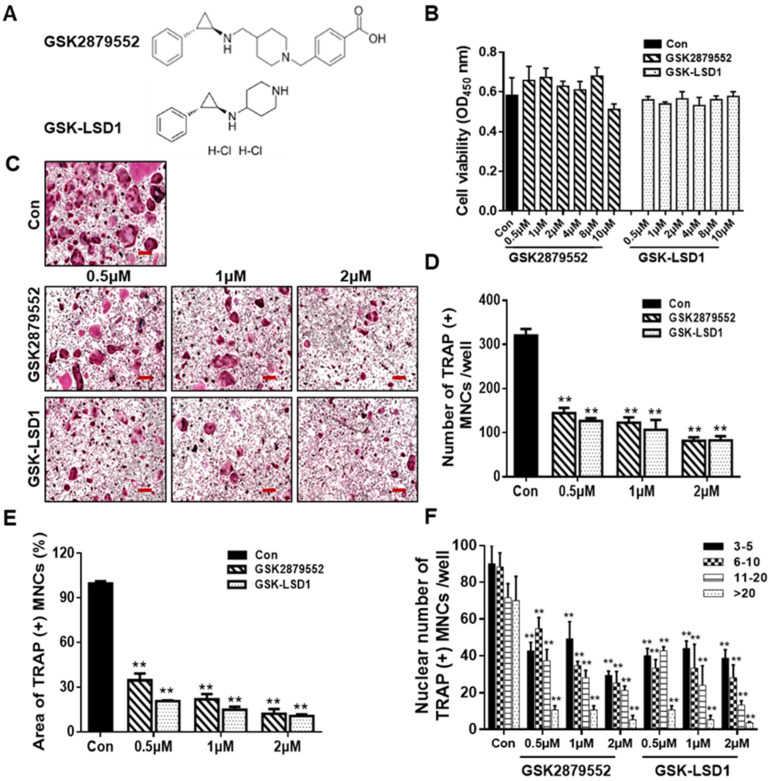
LSD1 inhibitors prevent RANKL-stimulated osteoclast differentiation in a dose-dependent manner. (**A**) Structure of GSK2879552 and GSK-LSD1. (**B**) BMMs were treated with or without LSD1 inhibitors for 48 h. Cell viability was confirmed by the EZ-cytotoxicity kit. OD, optical density. (**C**) BMMs were incubated with M-CSF and RANKL cultured at different dosages of LSD1 inhibitors (0.5, 1, and 2 μM) for 4 days, and stained with the TRAP staining kit. Scale bar = 200 µm. (**D**,**E**) Average area and number of TRAP (+) MNCs in the three wells using Image J software. (**F**) Nuclei number of TRAP (+) MNCs were analyzed using Image J. Data are presented as mean ± SD of three independent experiments; ** *p* < 0.01 versus the control group.

To assess the effect of inhibition of LSD1 during osteoclastogenesis, we treated BMMs with LSD1 inhibitors, GSK2879552 and GSK-LSD1, and examined their effects on osteoclast differentiation. The chemical structures of GSK2879552 and GSK-LSD1 are shown in Figure 2A. Cell viability assay was used to examine the effects of LSD1 inhibitors on the cellular activity of BMMs. As shown in Figure 2B, LSD1 inhibitors did not significantly affect the viability of BMMs. TRAP staining was performed to further examine the influence of LSD1 inhibitors on the formation of osteoclasts. The cells were cultured with RANKL and various concentrations of LSD1 inhibitors (0.5, 1, and 2 μM) for 4 days. Consistent with the knockout LSD1 results, the LSD1 inhibitors prevent osteoclast differentiation in a dose-dependent manner (Figure 2C). Statistical analysis demonstrated that LSD1 inhibitors effectively suppressed the size, number, and nuclei of TRAP-positive multinucleated cells (Figure 2D–F). Hence, LSD1 inhibitors can reduce osteoclast differentiation without any cytotoxicity.

To further determine the influence of LSD1 inhibitors on osteogenic differentiation, calvarial cells were stimulated with BMP2 in the presence or absence of LSD1 inhibitors for osteoblast differentiation. As shown in Figure S1A, ALP staining showed that GSK2879552 did not inhibit osteoblast differentiation; however, GSK-LSD1 slightly inhibited osteoblast formation at a concentration of 2 μM. The intensity of ALP staining is visible in Figure S1B. These results suggested that GSK2879552 suppressed osteoclast differentiation without affecting osteoblast formation at the indicated concentrations. GSK-LSD1 slightly inhibited osteoblast differentiation at a concentration of 2 μM.

### 2.3. Suppression of RANKL-Stimulated F-Actin Rings and Bone Resorption by LSD1 Inhibitors

**Figure 3:**
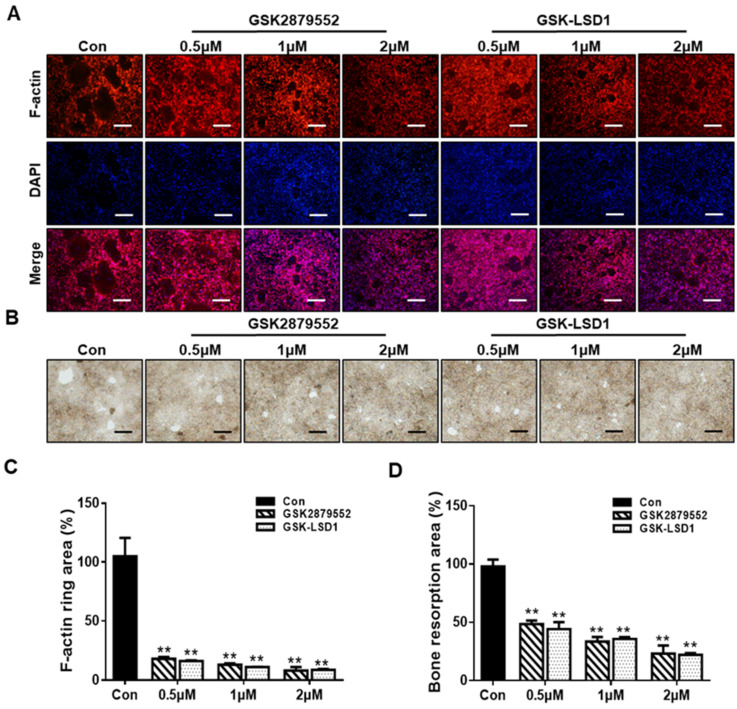
LSD1 inhibitors suppress RANKL-stimulated F-actin rings and bone resorption. (**A**) BMMs were incubated with MCSF and RANKL in the presence or absence of LSD1 inhibitors for 5 days. The cells were fixed and stained with rhodamine-conjugated phalloidin. Scale bar = 200 µm. (**C**) Area of actin rings was calculated by Image J. (**B**) Representative figure of bone resorption pits in each group. BMMs were seeded into bone resorption assay kit 48 plates and treated with or without LSD1 inhibitors for 5 days. Scale bar = 200 µm. (**D**) Pit area was measured with image J. The data presented are the mean ± SD of three independent experiments. ** *p* < 0.01 versus the control group.

We further explore the effect of LSD1 inhibitors on F-actin ring formation and bone resorption during osteoclastogenesis. LSD1 inhibitors significantly disrupted F-actin ring formation, compared to the control (Figure 3A). As shown in Figure 3C, the size of an F-actin ring is significantly reduced by LSD1 inhibitors. In addition, the bone-resorption results showed the presence of many bone-resorption pits in the control group, while the number of these bone-resorption pits is lower in the LSD1-inhibitor-treatment groups (Figure 3B,D). Hence, both F-action formation and bone resorption were inhibited by LSD1 inhibitors in vitro.

### 2.4. LSD1 Inhibitors Reduce the Expression of Osteoclast-Specific Genes and Proteins

**Figure 4:**
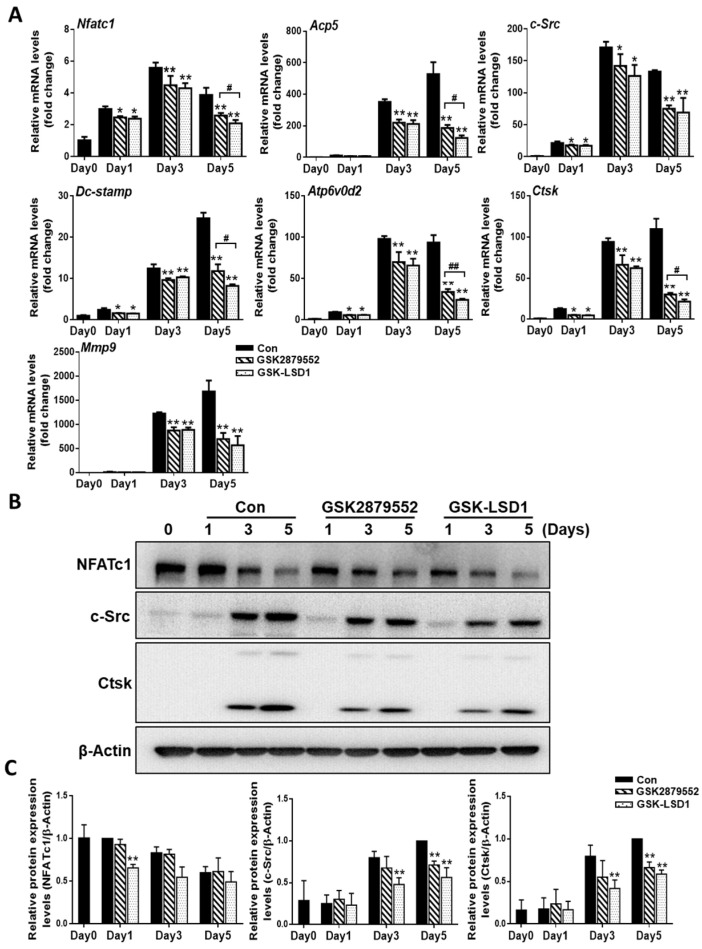
LSD1 inhibitors decrease the expression of osteoclast-specific genes and proteins. BMMs were cultured with or without LSD1 inhibitors for 1, 3, and 5 days. (**A**) The mRNA expression of osteoclast-related genes was analyzed by qRT-PCR. The mRNA was standardized to GAPDH control. The data presented are the mean ± SD of three independent experiments. * *p* < 0.05; ** *p* < 0.01, LSD1 inhibitors treatment group compared with the control group. ^#^ *p* < 0.05; ^##^ *p* < 0.01, the GSK2879552 treatment group compared with the GSK-LSD1 treatment group. (**B**) Western blotting analysis was performed to indicate the expression of osteoclast-specific proteins. ** *p* < 0.01, LSD1 inhibitors treatment group compared with the control group. (**C**) The protein levels of NFATc1, c-Src, and Ctsk were calculated by image J, and normalized to β-Actin.

To evaluate the molecular mechanisms involved in the inhibition of osteoclastogenesis by LSD1 inhibitors, we confirm the mRNA expression of osteoclast-specific genes by RT-PCR, including *Acp5, Mmp9, Dc-stamp, c-Src, Atp6v0d2, Ctsk*, and *Nfatc1* (Figure 4A). Our results indicated that the relative mRNA expression of osteoclast-specific genes was significantly suppressed with LSD1 inhibitors on day 5. We further investigated the expression of osteoclast-specific proteins during osteoclast differentiation in the presence or absence of LSD1 inhibitors. As shown in Figure 4B, the protein expression of c-Src and Ctsk was significantly decreased by LSD1 inhibitors in the late stage of osteoclast differentiation. However, the protein expression of NFATc1 was significantly reduced by GSK-LSD1 on day 1. Hence, LSD1 inhibitors decrease the expression of osteoclast-specific genes and proteins during osteoclast differentiation.

### 2.5. LSD1 Inhibitors Reduce the Demethylation Activity of LSD1

**Figure 5:**
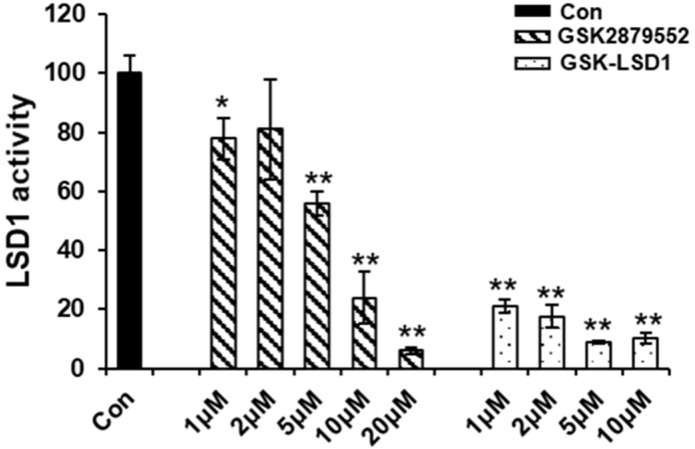
Effect of LSD1 inhibitor demethylation activity of LSD1. The effect of LSD1 inhibitors on LSD1 activity was determined by the LSD1 inhibitor screening kit. The data presented are the mean ± SD of three independent experiments. * *p* < 0.05; ** *p* < 0.01, compared with the control group.

To examine the effect of LSD1 inhibitors on the LSD1 activity during osteoclast differentiation, we checked the demethylation activity of LSD1 with small-molecule inhibitors. LSD1 not only removes methyl groups from mono-methylated lysine 4 and lysine 9 on histone H3, but also removes them from di-methylated lysine 4 and lysine 9 on histone H3 [32]. The results of the LSD1 activity assay indicated that the demethylation activity of LSD1 was significantly suppressed by GSK-LSD1, and only dramatically inhibited at high concentrations of GSK2879552 (Figure 5). Hence, GSK2879552 and GSK-LSD1 decreased the demethylation activity of LSD1. However, the LSD1-inhibitor treatment during osteoclast differentiation did not cause any significant difference in the protein expression of LSD1 and mono- and di-methylation of histone H3 lysine K4 and lysine K9 (Figure S2).

### 2.6. Knockout of LSD1 in Raw 264.7 Cells Suppresses RANKL-Induced Osteoclast Differentiation

**Figure 6:**
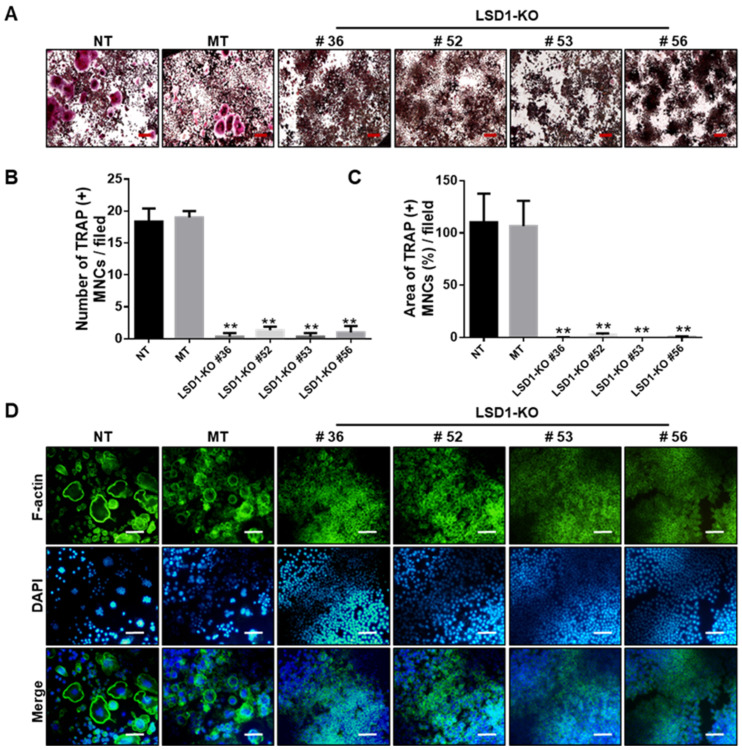
LSD1 deficiency suppresses RANKL-induced osteoclast differentiation and F-actin ring formation. (**A**) TRAP staining was performed to determine differentiated osteoclasts from transfected Raw 264.7 cells. Representative images of TRAP staining are shown. Scale bar = 200 µm. (**B**,**C**) Number and area of TRAP (+) MNCs were calculated from three randomly selected images in each group by Image J software. ** *p* < 0.01 versus the MT group. (**D**) Transformed Raw 264.7 cells were incubated with RANKL (50 ng/mL) for 5 days. Cells were stained with FITC-conjugated phalloidin and pictures were captured by the fluorescence microscope. Scale bar = 100 µm. NT: no transfection, MT: mock transfection.

To further determine the role of LSD1 during osteoclast differentiation, Raw 264.7 cells were incubated with RANKL, and the protein expression of LSD1 was detected by Western blot (Figure S3A). The LSD1 protein expression increased at the middle stage of osteoclast differentiation in Raw 264.7 cells. Next, we knockout LSD1 in Raw 264.7 cells using the CRISPR/Cas9 system to further elucidate the role of LSD1 in osteoclast differentiation. Based on the results of targeted deep sequencing, we selected five Raw 264.7 cell clones (Figure S3D) for further characterization. The mock transfection (MT) #21 clone was used as the vector control. Protein and gene levels of the LSD1 of all clones (MT #21, KO #36, KO #52, KO #53, and KO #56) and no-transfection (NT) cells were examined by Western blot and RT-PCR, respectively. These results indicated that the knockout of LSD1 was successful (Figure S3B,C). Next, we examined the effects of LSD1 knockout in RANKL-induced osteoclast differentiation. As shown in Figure 6A, RANKL-stimulated osteoclast formation was significantly attenuated by LSD1 knockout. Quantitative analysis of TRAP staining results indicated that the area and numbers of the nuclei of TRAP-positive cells (≥3 nuclei per cell) in the LSD1 knockout group were significantly lower than those in the Raw 264.7 cells of the NT and MT group (Figure 6B,C). In addition, we examined the effect of LSD1 knockout on F-actin ring formation. Consistent with the results of the TRAP staining assay, LSD1 knockout could significantly reduce the formation of F-actin rings (Figure 6D). Hence, LSD1 is a key regulatory factor of RANKL-induced osteoclast differentiation and F-actin ring formation.

### 2.7. GSK2879552 Prevents Cortical Bone Loss in Ovariectomized (OVX) Mice

**Figure 7:**
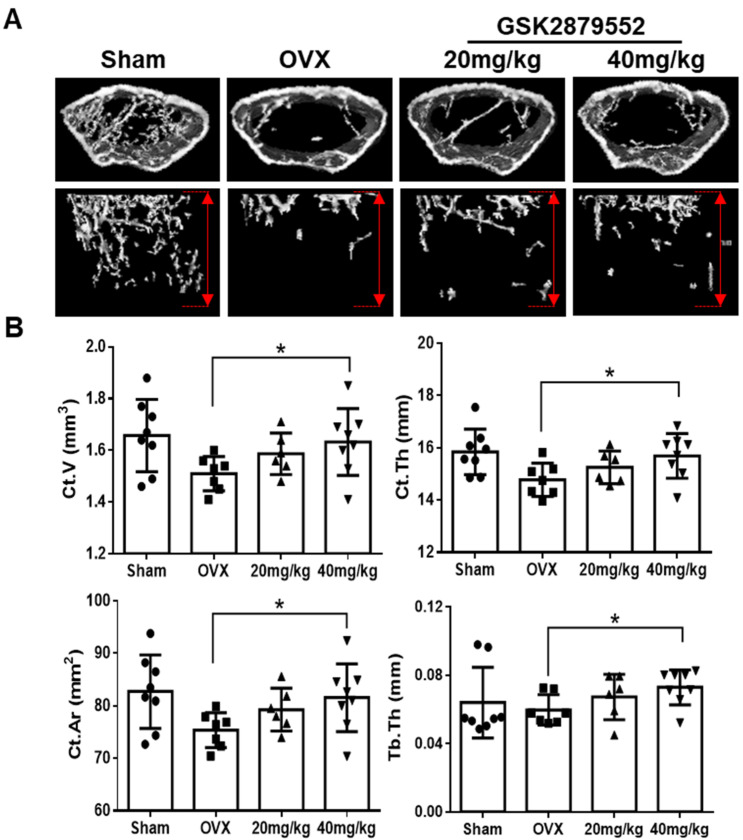
GSK2879552 suppressed OVX-induced osteoporosis in mice. (**A**) Fixed femurs of mice were analyzed by micro-computed tomography in the Sham, OVX, GSK-2879552 (20 mg/kg and 40 mg/kg) treatment groups. Three-dimensional reconstructed images of the cortical bone (**top**) and the trabecular bone (**bottom**) of mice femurs are shown. The length of the *y*-axis of the area of analysis was marked by a red double arrow, which indicates 2 mm below the epiphyseal plate of the bone in each group. (**B**) Ct.V, Ct.Th, Ct.Ar, and Tb.Th were measured for each sample. Values are expressed as mean ± SD. Ct.V, cortical bone volume; Ct.Th, cortical bone thickness; Ct.Ar, cortical bone area; Tb.Th, trabecular bone thickness; * *p* < 0.05 vs. the OVX group.

Because GSK2879552 inhibited RANKL-induced osteoclast differentiation in vitro, we examined the anti-osteoporotic effects of GSK2879552 in vivo using ovariectomized (OVX) mice. After ovariectomy, the OVX mice were treated with different concentrations of GSK2879552 via intraperitoneal injection. After 4 weeks of treatment, the mice were sacrificed and their femurs were analyzed using micro-computed tomography (μCT). Successful OVX was confirmed by a marked decrease in uterine weight (Figure S4A,B). No obvious change in the body weight of OVX and GSK2879552-treatment groups was noted (Figure S4C), indicating that GSK2879552 has no toxic effects. The μCT images are shown in Figure 7A. The structural parameters of the distal femur showed that the cortical bone loss reduced under high doses of GSK2879552 compared with that in the OVX group, as shown by increased Ct.V, Ct.Th, Ct.Ar, and Tb.Th (Figure 7B). However, when high doses of GSK2879552 were used, BMD was not restored compared to that in the OVX group (Figure S4D). Hence, GSK2879552 protects against OVX-induced cortical bone loss.

## 3. Discussion

Epigenetic regulators have received attention as targets for the treatment of several diseases, such as cancer [33] and acute myeloid leukemia [34]. Epigenetic factors can influence bone remodeling. They also play an important role in osteoclast differentiation [11]. Previous studies have also shown that histone methylation is regulated by histone methyltransferases and histone demethylases, which in turn are involved in the regulation of bone cell differentiation [18]. Because epigenetic regulators can influence bone remodeling, they are potential targets for osteoclast differentiation. Among the 16 small-molecule inhibitors used in this study, LSD1 inhibitors showed the highest inhibitory effect (Figure 1).

LSD1, a histone methyl-modification enzyme, plays a key role in various physiological and pathological processes through its demethylase activity with both histone and non-histone targets. LSD1 is involved in bone remodeling, including osteoblast differentiation of human mesenchymal stem cells [28]. However, the function of LSD1 during the RANKL-mediated osteoclast formation is not clear. Therefore, in this study, we focused on investigating the role of LSD1 in RANKL-induced osteoclast differentiation of murine macrophages by LSD1 inhibitors and the knockout of LSD1 in Raw264.7 cells. It has previously been described that GSK-LSD1 is a structurally similar compound to GSK2879552, which is also a selective irreversible in-activators of LSD1 [35]. Thus, we employ both LSD1 inhibitors, GSK-LSD1 and GSK2879552, to check the effects of LSD1 inhibitors on osteoclast formation. TRAP staining results indicate that LSD1 inhibitors suppress RANKL-mediated osteoclast formation (Figure 2). Meanwhile, our ALP staining results suggested that LSD1 inhibitors suppress osteoclast differentiation and do not affect osteoblast formation at the indicated concentrations. Moreover, bone resorption is one of the major characteristic features of aberrant osteoclast activation, which is regulated by the formation of actin rings [36,37]. In this study, we also indicated that LSD1 inhibitors suppress RANKL-induced bone resorption and F-actin ring formation (Figure 3). The differentiation of osteoclasts is complex progress and requires cell–cell fusion. DC-STAMP plays a major role in regulating this process [38]. Additionally, several other critical markers, including *Nfatc1*, *Oc-stamp*, *Mmp9*, *Atp6v0d2*, and *Ctsk*, have been shown to be involved at different stages of the osteoclast-differentiation process [39]. Therefore, we examined the effect of LSD1 inhibitors on the mRNA expression of osteoclast-related markers. Our data suggested that osteoclast differentiation-related gene expressions were suppressed by LSD1 inhibitors (Figure 4A). In osteoclast differentiation, activation of c-Src by RANKL plays a crucial role in the regulation of osteoclast function [40]. Ctsk is secreted by differentiated osteoclasts to degraded collagen and matrix proteins during bone resorption [41]. In the present study, our results suggested LSD1 inhibitors inhibited the expression of c-Src, and Ctsk in RANKL-induced osteoclastogenesis (Figure 4B). These results indicated that LSD1 inhibitors attenuate osteoclast activity. NFATc1 protein expression was significantly inhibited by GSK-LSD1 in osteoclast differentiation, and GSK2879552 weakly decreased NFATc1 expression, however, it is no significant difference compared with the control group. It is not clear whether methylation of NFATc1 is involved in osteoclast differentiation through the LSD1 activity.

To further investigate the function of LSD1 during osteoclast differentiation, we used TRAP staining and F-actin ring staining to investigate the influences of the deletion of LSD1 on RANKL-induced osteoclast differentiation and F-actin ring formation, respectively. Knockout of LSD1 in Raw 264.7 cells showed impaired osteoclast formation in TRAP-positive osteoclasts, and F-actin ring formation (Figure 5A,B), which is similar to the effect of LSD1 inhibitors. These results have been supported by a previous study that showed that the inhibition of LSD1 with LSD1-specific inhibitors and small interfering RNAs in human osteoclast precursors represses osteoclast differentiation [42]. Our results confirmed that LSD1 was a positive regulator in RANKL-induced osteoclastogenesis of BMMs, and knockout LSD1 significantly reduced osteoclast formation. Hence, LSD1 could be used as a therapeutic target for the treatment of excessive osteoclast-mediated bone diseases.

LSD1 is an eraser enzyme regulating the methylation of lysine 4 and lysine 9 on histone H3 [32]. To further examine the effects of LSD1 inhibitors on the LSD1 demethylase activity during osteoclast differentiation, we investigated the demethylation activity of LSD1 after the LSD1-inhibitor treatment. The results of the LSD1 activity assay indicated that both GSK2879552 and GSK-LSD1 decreased the demethylation activity of LSD1. Astleford-Hopper et al. also showed that the methylation of H3K4 increased in the LSD1cKO mice [30]. However, our result suggested the global methylation of H3K4 and H3K9 was not regulated by LSD1 inhibitors during RANKL-mediated osteoclast differentiation. A recent report showed that LSD1 knockdown did not affect global H3K4 methylation levels, genome-wide ChIP seq analysis revealed high levels of LSD1 at gene promoters, and its binding was associated with di- and tri-methylation of histone 3 at lysine 4 in osteoblastogenesis [29]. Hence, LSD1 inhibitors may attenuate RANKL-induced osteoclast differentiation by affecting the methylation activity of specific genes promoter. There are some limitations to this result that warrant further investigation.

To indicate the effect of LSD1 inhibitors on bone loss, OVX model mice were generated and intraperitoneally injected with 10 mg/kg of GSK2879552 and 10 mg/kg of GSK-LSD1 or vehicle (DMSO) for four weeks. The micro-CT results suggested that both compounds did not markedly suppress the OVX-induced bone loss. Next, we increased the dose of GSK2879552 administration in mice. After the OVX surgery, the mice were injected with vehicle (DMSO) or GSK2879552 (20 mg/kg; 40 mg/kg). The morphometric analysis results demonstrated that GSK2879552 significantly recover the Ct.V, Ct.Th, Ct.Ar, and Tb.Th of a mouse, compared to that of the OVX group; however, it does not have any significant effect on BMD. Previous reports showed that the protection of cortical bone mass by estrogens is mediated [43]. And LSD1 was reported to control the coordinated expression of genes responsive to estrogens [19]. Hence, GSK2879552 impacts the cortical bone loss of the OVX-induced osteoporosis mice model might by regulating the estrogens.

In conclusion, our results elucidate that LSD1, as a positive regulator, is involved in regulating RANKL-induced osteoclast differentiation. Furthermore, we suggest that LSD1 inhibitors GSK2879552 and GSK-LSD1 can repress osteoclast formation and bone resorption in vitro. Thus, pharmacological inhibition of LSD1 could improve the treatment of osteoporosis.

## 4. Materials and Methods

### 4.1. Materials and Reagents

Eagle minimal essential medium-alpha modification (α-MEM) and fetal bovine serum (FBS) were purchased from Thermo Fisher Scientific (Waltham, MA, USA). M-CSF and RANKL were procured from PeproTech EC, Ltd. (Cranbury, NJ, USA). Tartrate-resistant acid phosphatase staining kit was obtained from CosmoBio (Tokyo, Japan). Antibodies for NFATc1 (#8032s) and Cathepsin K (#48353), LSD1 (#2139), Mono-Methyl-Histone H3(Lys4) (#9723), and Mono-Methyl-Histone H3(Lys9) (#14186) were purchased from Cell Signaling Technology (Beverly, MA, USA), and β-actin antibody was purchased from Sigma-Aldrich, Inc. (St. Louis, MO, USA). GSK2879552 (molecular formula: C_23_H_28_N_2_O_2_, molecular weight: 364.5, PubChem CID: 66571643) was purchased from Selleckchem (Houston, TX, USA). GSK-LSD1 dihydrochloride (molecular formula: C_14_H_22_Cl_2_N_2_, molecular weight: 289.24, PubChem CID: 91663353) was purchased from MedChemExpress (Princeton, NJ, USA). Other epigenetic regulator inhibitors (JQ-1, ABBV-744, EPZ015866, Vorinostat, Remodelin, Panobinostat, Belinostat, Selisistat, C646, A-366, PFI-2, Tazemetostat, GSK-J4, JIB-04, and Pinometostat) were purchased from ChemScene (Monmouth Junction, NJ, USA), were dissolved in dimethyl sulfoxide (DMSO; Sigma-Aldrich, St. Louis, MO, USA). The structures of epigenetic regulation inhibitors corresponding to these numbers are shown in Table S1.

### 4.2. Osteoclast and Osteoblast Culture

Bone marrow macrophages (BMMs) were isolated from the 10-week-old C57BL6 mice. The primary osteoclast cells were grown in α-MEM without nucleosides containing 10% FBS and 1% penicillin-streptomycin solution supplemented with 30 ng/mL of M-CSF for 3 days. The adherent cells were harvested and used as osteoclast precursors. The primary osteoblasts were isolated from the calvarial bones of 3-day baby mice. The primary osteoblast was cultured in α-MEM containing 10% chFBS and 1% penicillin-streptomycin solution. All the cells were incubated at 37 °C with a humidified 5% CO_2_ atmosphere.

### 4.3. TRAP Staining Assay

BMMs were seeded in 96-well plates at a density of 1 × 10^4^ cells/well in complete α-MEM containing 30 ng/mL of M-CSF. After 1 day, the cells were treated with RANKL (50 ng/mL) in the absence or presence of various concentrations of LSD1 inhibitors to induce differentiation into osteoclasts. In addition, to confirm the effect of LSD1 on osteoclast differentiation, we used the knockout LSD1 gene in Raw 264.7 cells using the clustered regularly interspaced short palindromic repeat-Cas9 genome-editing system (CRISPR-Cas9 system). No transfected cells and LSD knockout cells were also stimulated with RANKL to induce differentiation. The differentiated cells were fixed with 4% paraformaldehyde (PFA) solution for 20 min at room temperature and then washed with PBS. Next, the cells were stained for TRAP following the manufacturer’s instructions. Finally, the stained samples were imaged by a microscope. The area and number of TRAP-positive osteoclasts were calculated using the Image J software (1.8.0_112 version, National Institutes of Health, Bethesda, MD, USA).

### 4.4. ALP Staining

The primary osteoblast cells were treated with or without LSD1 inhibitors in the presence of BMP2 (100 ng/mL) for 7 days. Media were refreshed every two days. The cells were washed with PBS, fixed with 70% ice-cold ethanol for 30 min, and rinsed with double-distilled H_2_O twice. Cells cultured for 7 days were used for ALP staining. The cells were stained with the BCIP^®^/NBT Liquid Substrate System (Sigma Aldrich, St. Louis, MO, USA) for 20 min at RT following the manufacturer’s instructions. The images were captured with a microscope. The intensity of ALP staining was quantified by Image J software.

### 4.5. Cytotoxicity Assay

The cell viability was determined as described previously [44]. Briefly, BMM cells were seeded in 96-well plates for 24 h. Then, cells were treated with M-CSF in the presence or absence of various concentrations of LSD1 inhibitors for 2 days. The cell viability was tested by EZ-Cytox Kit (Dogen Bio, Kyoto, Japan) according to the manufacturer’s manual. Finally, the OD value was determined at 450 nm by using a microplate reader (San Jose, CA, USA).

### 4.6. F-Actin Ring Staining

F-actin ring formation is a critical characteristic of mature cytoskeletal in osteoclasts [36]. For F-actin ring staining, BMMs were seeded in 12-well plates and stimulated with M-CSF, RANKL, and indicated with or without various concentrations of LSD1 inhibitors for 5 days. Raw 264.7 cells of no-transfected or LSD1 knockout were also stimulated with RANKL to induce osteoclast differentiation. The differentiated cells were fixed with 4% PFA solution for 20 min at room temperature. The fixed cells were permeabilized with 0.1% Triton-X 100 and blocked with 2% BSA for 1 h. The cells were incubated with rhodamine-conjugated phalloidin for 2 h and washed with PBS. Finally, the cells were incubated with DAPI (4’,6-diamidino-2-phenylindole). The images were captured by fluorescence microscopy and analyzed by Image J Software.

### 4.7. Bone Resorption Assay

Bone resorption was assessed by the bone resorption assay kit (Cosmo Bio Co., Ltd., Tokyo, Japan), as previously described [45]. The BMMs were seeded into a 48-well bone resorption assay plate at a density of 2.5 × 10^4^ cells/well. The cells were differentiated with M-CSF and RANKL in the absence or presence of indicated concentrations of LSD1 inhibitors. After cell differentiation, the attached cells were removed using 5% sodium hypochlorite. The plates were air-dry at RT and resorption pits were captured by a microscope. In addition, the resorption areas were quantified by analyzing three randomly selected pictures per well by Image J software.

### 4.8. Real-Time PCR Assay

Total RNA was extracted from cells with the TRIzol reagent (Qiagen Sciences, Valencia, CA, USA) according to the manufacturer’s instructions. RNA concentrations were quantified, and then reversely transcribed using PrimeScript RT Reagent Kit (Takara Biotechnology, Shiga, Japan) with 500 ng RNA per reaction. Next, the reaction mixture was incubated at 37 °C for 30 min and 85 °C for 15 s, and then stored at 4 °C. Real-time PCR amplification reactions were performed by Power SYBR Green PCR Master Mix and appropriate primers in a volume of 20 μL. The mouse glyceraldehyde-3-phosphate dehydrogenase (GAPDH) gene was used as the reference gene. The primer sequence is presented in Table S2.

### 4.9. Western Blot Assay

The BMM cells were stimulated with RANKL and treated with or without LSD1 inhibitors for 1, 3, or 5 days. The cells were washed with PBS, harvested by a cell scraper, and lysed in the radioimmuno-precipitation buffer. The supernatant was collected following sonication and centrifugation. The concentrations of the proteins were determined using the BCA protein assay following the manufacturer’s protocol. Proteins from each lysate sample were separated by electrophoresis using the polyacrylamide gel electrophoresis (PAGE) gel and transferred to polyvinylidene difluoride membranes (Bio-Rad Laboratories, Hercules, CA, USA). The membranes were blocked with 5% non-fat dry milk in TBST for 1 h, and then incubated overnight with primary antibodies at 4 °C. The next day, the membranes were washed with PBS and incubated for 2 h with secondary antibodies. Immunoreactive protein bands were detected by the ECL Western Blotting Detection Reagent (Amersham, Buckinghamshire, UK).

### 4.10. LSD1 Activity Analysis

The effect of LSD1 inhibitors on the LSD1 activity was detected by the LSD1 inhibitor screening kit according to the manufacturer’s instructions (Caymanchem, MI, USA). Firstly, a sample mixture, assay buffer, LSD1 human recombinant assay reagent, LSD1 assay fluorometric substrate, LSD1 assay horseradish peroxidase, and LSD1 assay peptide were added to a 96-well plate (black). Background wells did not add LSD1 assay peptide, 100% initial activity well not added inhibitors, and inhibitor wells added several concentrations of GSK2879552 and GSK-LSD1. Secondly, the plate was covered and incubated at 37 °C for 30 min. The fluorescence was measured using an excitation wavelength of 530–540 nm and an emission wavelength of 585–595 nm by the SpectraMax i3x multi-mode microplate detection platform (Molecular Devices, Silicon Valley, CA, USA). Finally, the inhibition rate was calculated as follows: % Inhibition = [(Initial activity − Sample)/Initial activity] × 100.

### 4.11. Generation of LSD1-Knockout Raw 264.7 Cells by the CRISPR-Cas9 System

Firstly, we designed the guide sequences for targeting a gene. The mouse guide sequence for LSD1 was as follows: 5′-CCGAGACCCCGGAGGGCCGACGG-3′, followed by synthesis and purification of sgRNA in vitro. Secondly, the sgRNA and Cas9 proteins were delivered into Raw 264.7 cells by Electric Transfection. After 3 days, the transfected cells were separated into 96-well plates for making a single clone. Finally, the initial identification of the knockout clones was carried out by targeted-deep sequencing.

### 4.12. OVX-Induced Osteoporosis Mice Model and Micro-Computed Tomography

C57BL/6J mice were purchased from DAMOOL SCIENCE. All mice were divided into four groups: Sham group (*n* = 8), ovariectomy (OVX) group (*n* = 7), OVX with a low dose of the GSK2879552 group (20 mg/kg, *n* = 6), or high-dose GSK2879552 group (40 mg/kg, *n* = 8). For the OVX operation, the mice underwent bilateral ovariectomy. The sham-group mice were handled similarly; however, their ovaries were not removed. One week after surgery, the mice were injected intraperitoneally every day for four weeks. The body weights of all mice were measured weekly. Finally, the mice were sacrificed and their femur and tibia bones were collected. The fixed femurs were analyzed by using a Quantum GX μCT imaging system (PerkinElmer, Hopkinton, MA, USA) at the Korea Basic Science Institute (Gwangju, Korea). The scan conditions for μCT analysis were as described in a previous study [46].

### 4.13. Statistical Analysis

All data in vitro experiments presented are the mean ± standard deviation (SD) of three independent experiments. The significant differences between the control and experimental groups were determined by a two-tailed Student’s *t*-test. The data were analyzed using Excel. Quantitative analysis graphs were obtained by Excel (Microsoft Corporation, Redmond, WA, USA) or GraphPad Prism 6.0 (GraphPad Software Inc., San Diego, CA, USA). * *p* < 0.05, ** *p* < 0.01, ^#^ *p* < 0.05, and ^##^ *p* < 0.01 were considered significant differences.

## Data Availability

The raw data supporting the findings of this study will be made available by the authors, without undue reservation, once the article is accepted.

## Supplementary Materials

The following supporting information can be downloaded at: https://www.mdpi.com/article/10.3390/ijms24043605/s1.
