# Supplementary material for: Crucial Role of Lysine-Specific Histone Demethylase 1 in RANKL-Mediated Osteoclast Differentiation
Source: Int J Mol Sci. 2023 Feb 10;24(4):3605. doi: 10.3390/ijms24043605 (PMC9967819; doi:10.3390/ijms24043605)
Supplement: Supplementary file 1 [file ijms-24-03605-s001.zip › ijms-2138755-supplementary.pdf]

International Journal of *Molecular Sciences*

MDPI

Supplementary Materials

Crucial Role of Lysine-Specific Histone Demethylase 1 in RANKL-Mediated Osteoclast Differentiation

Mina Ding [1], Zhihao Chen [1], Eunjin Cho [2], Sang-Wook Park [2,*] and Tae-Hoon Lee [2,*]

[1] BioMedical Sciences Graduate Program (BMSGP), Chonnam National University, Gwangju 61186, Republic of Korea; minading1021@gmail.com (M.D.); chinaczhihao@gmail.com (Z.C.)

[2] Department of Oral Biochemistry, Dental Science Research Institute, School of Dentistry, Chonnam National University, Gwangju 61186, Republic of Korea; ag8414@gmail.com

* Correspondence: swpark@chonnam.ac.kr (S.-W.P.); thlee83@jnu.ac.kr (T.-H.L.)

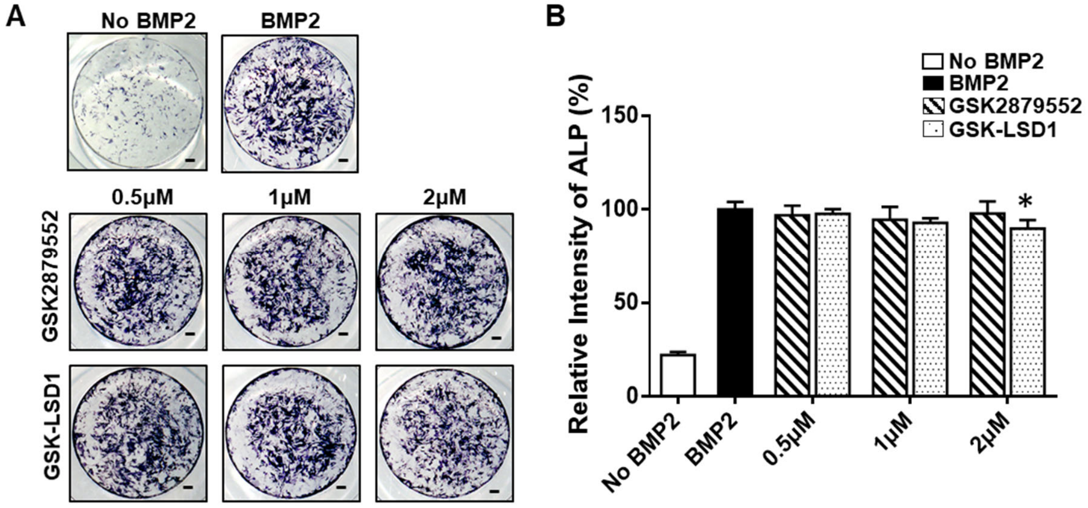

Figure S1. Effect of LSD1 inhibitors on BMP2-induced osteoblast formation. (**A**) Mouse (C57BL6J) calvarial cells were incubated with BMP2 (100 ng/mL) in the presence or absence of LSD1 inhibitors with indicated concentration for 7 days. Cells were fixed and stained with ALP. Representative images of ALP staining. Scale bar represents 400 μm. (**B**) Relative intensity of ALP staining were quantified by Image J. * $P < 0.05$ versus the BMP2 group.

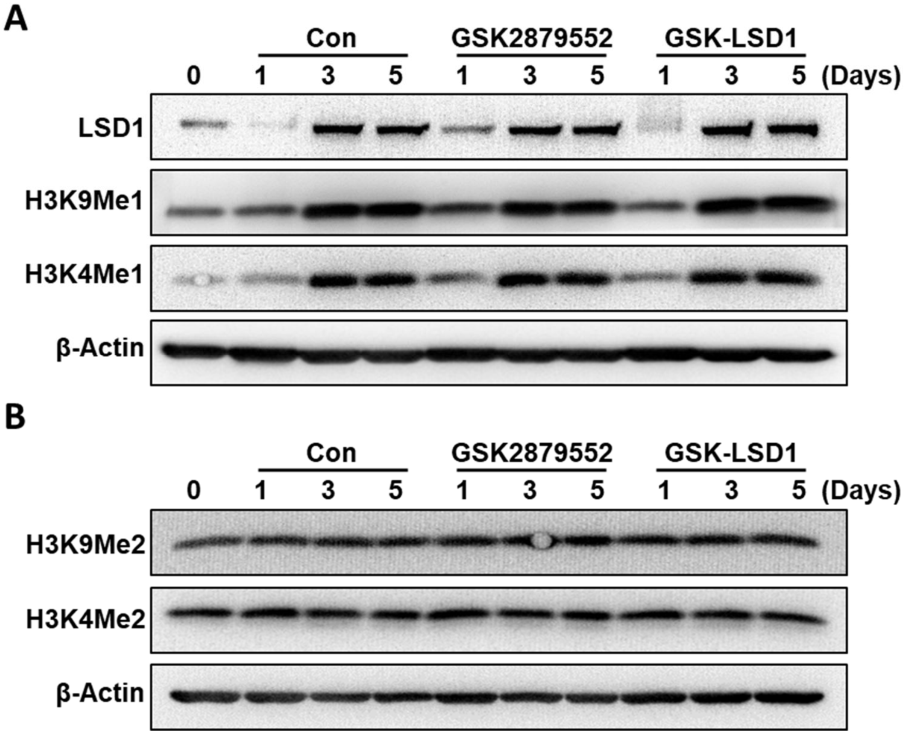

Figure S2. Effect of LSD1 inhibitors on LSD1 protein expression and Histone 3 methylation. (**A**,**B**) BMMs were induced with M-CSF and RANKL in the presence or absence of LSD1 inhibitors for 1, 3 and 5 days, respectively. Proteins were extracted, and the protein expression of LSD1, H3K4me1, H3K9me1, H3K4me2 and H3K9me2 were performed by Western blotting.

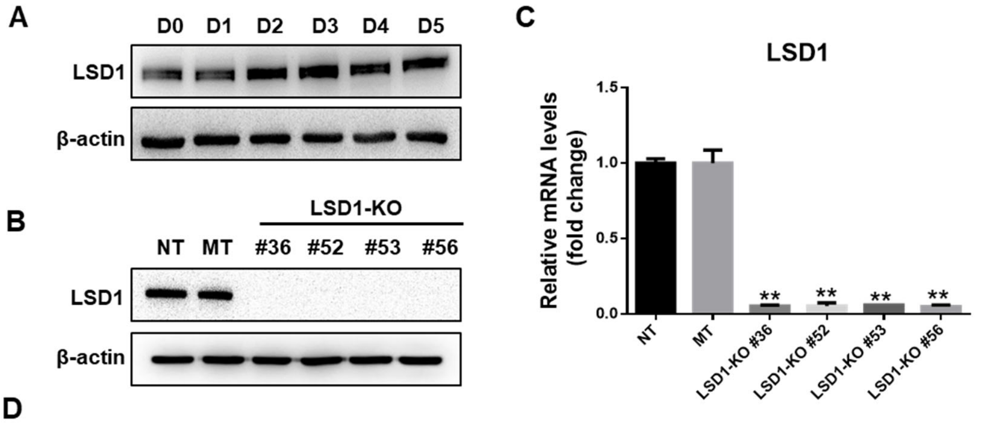

D

Deep sequence results

Clone No.	Total read	Ins read	Del read	Indel rate (%)
MT(#21)	5267	0	13	13 (0.2%)
KO(#36)	5212	0	5210	5210 (100.0%)
KO(#52)	6339	0	6339	6339 (100.0%)
KO(#53)	7217	2940	4272	7212 (99.9%)
KO(#56)	7119	0	7119	7119 (100.0%)

```
MT(#21) TCCGCGACGCCCATGGAGACCGGAATAGCCGAGACCCCGGAGGGCCGACGGACCAGCCGGCGCAAGCGG

KO(#36) TCCGCGACGCCCATGGAGACCGGAATAGCCGAGACCCCGGAGGG-CGACGGACCAGCCGGCGCAAGCGG (-1bp)

KO(#52) TCCGCGACGCCCATGGAGACCGGAATAGCCGAGACCCCGGAGGG-CGACGGACCAGCCGGCGCAAGCGG (-1bp)
        TCCGCGA-----------------------------------------------------CGCAAGCGG (-53bp)
        TCCGCGACGCCCATGGAGACCGGAATAGCCGAGACC--------CCGACGGACCAGCCGGCGCAAGCGG (- 8bp)
        TCCGCGACGCCCATGGAGACCGGAATAGC-------------------CGGACCAGCCGGCGCAAGCGG (-19bp)

KO(#53) TCCGCGACGCCCATGGAGACCGGAATAGCCGAGACCCCGGAGGG-CGACGGACCAGCCGGCGCAAGCGG (-1bp)
        TCCGCGACGCCCATGGAGACCGGAATAGCCGAGACCCCGGAGGGCGCGACGGACCAGCCGGCGCAAGCGG(+1bp)
        TCCGCGACGCCCATGGAGACCGGAATAGCCGAGACCCCGGAGGG----CGGACCAGCCGGCGCAAGCGG (-4bp)

KO(#56) TCCGCGACGCCCATGGAGACCGGAATAGCCGAGACCCCGGAGGG-CGACGGACCAGCCGGCGCAAGCGG (-1bp)
```

Figure S3. Knockout LSD1 in Raw 264.7 cells using the CRISPR/Cas9 system. (**A**) Raw 264.7 cells were treated with or without RANKL (50 ng/mL) for 1, 2, 3, 4, and 5 days. Expressions of LSD1 were determined by Western blotting. (**B**) Raw 264.7 cells were transfected with CRISPR/Cas9 system for knockout LSD1. The knockdown efficiency of LSD1 was measured by Western blotting. NT, no transfection; MT, mock transfection. (**C**) RT-PCR was used to further confirm the knockdown efficiency. ** $P < 0.01$ versus the MT group. (**D**) Deep sequence result of transfection cell clones. Ins read, insertion read; Del read, deletion read; Indel rate, the rate of insertion-deletion mutations. The underlined sequence in MT is the guide sequence for LSD1.

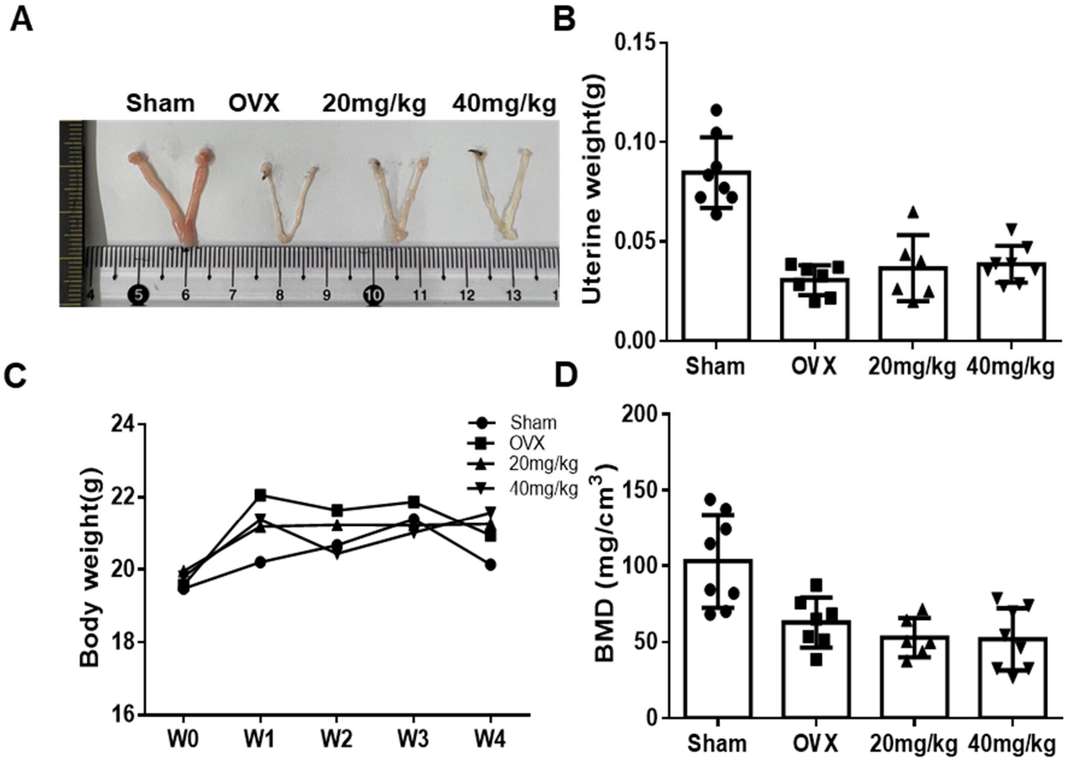

Figure S4. OVX-induced osteoporosis mouse model. (**A**) Representative uterine of the Sham, OVX, GSK2879552 treatment groups (20 mg/kg; 40 mg/kg). (**B**) Oviduct weight of mice in each group. (**C**) Mice body weight of the Sham, OVX, GSK2879552 treatment groups (20 mg/kg; 40 mg/kg). (**D**) Bone mineral density (BMD) of mice in each group.

Table S1. List of epigenetic regulators inhibitors for screening candidates.

**NO./Name**	**1. JQ-1 (carboxylic acid)**	**2. ABBV-744**	**3. EPZ015866**
**Structure**	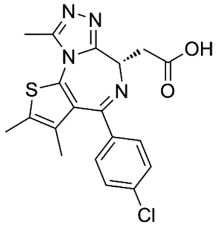	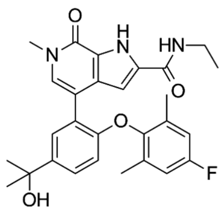	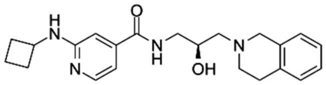
**NO./Name**	**4. Vorinostat**	**5. Remodelin (hydrobromide)**	**6. Panobinostat**
**Structure**	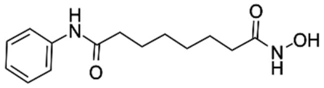	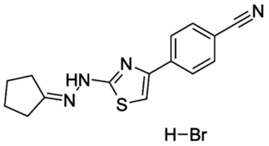	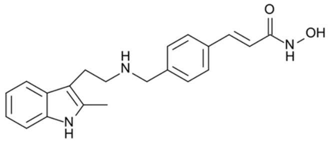
**NO./Name**	**7. Belinostat**	**8. Selisistat**	**9. C646**
**Structure**	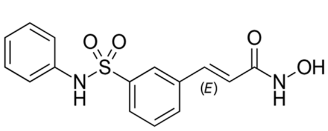	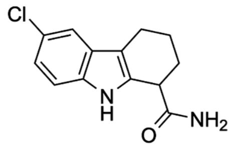	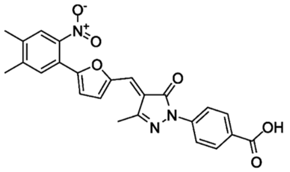
**NO./Name**	**10. A-366**	**11. PFI-2 (hydrochloride)**	**12. Tazemetostat**
**Structure**	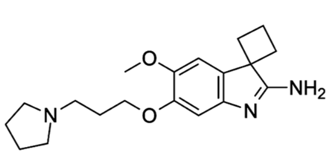	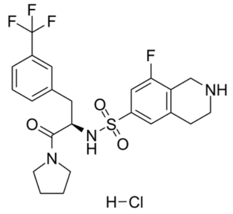	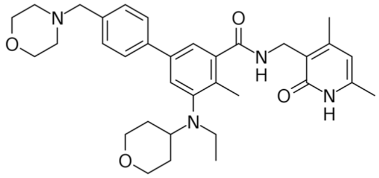
**NO./Name**	**13. GSK2879552**	**14. GSK-J4**	**15. JIB-04**
**Structure**	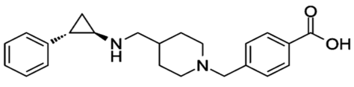	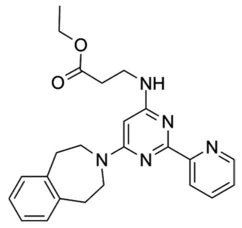	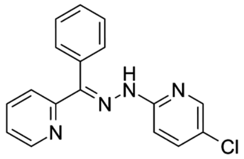
**NO./Name**	**16. Pinometostat**		
**Structure**	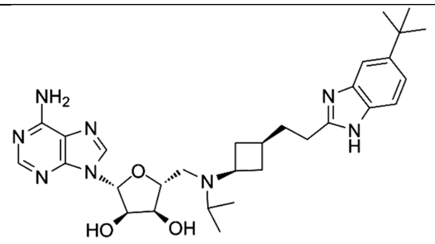		

Table S2. Primer sequences used for real-time PCR analysis.

Gene (mouse)	Primer sequence (5'-3')
Cathepsin k	F: ACTTCCGCAATCCTTACCGA<br>R: TTCGCTAGGCTCTTTTCGGA
Dc-stamp	F: CGCACGATGCTTCATTCTTC<br>R: CAGTGCCAGCCGCAATC
Oc-stamp	F: CAGAGTGACCACCTGAACAAACA<br>R: TGCCTGAGGTCCCTGTGACT
c-Src	F: CCAGGCTGAGGAGTGGTACT<br>R: CAGCTTGCGGATCTTGTAGT
Mmp9	F: CTGGACAGCCAGACACTAAAG<br>R: CTCGCGGCAAGTCTTCAGAG
Nfatc1	F: ACCACCTTTCCGCAACCA<br>R: GGTACTGGCTTCTCTTCCGTTTC
Gapdh	F: TGTGTCCGTCGTGGATCTGA<br>R: GATGCCTGCTTCACCACCTT
Lsd1	F: AGCGGGCCAAGGTAGAATACA<br>R: ATGGGGAAGTCGGCTTTGAAA
